# Activation of p53 by Nutlin-3a Induces Apoptosis and Cellular Senescence in Human Glioblastoma Multiforme

**DOI:** 10.1371/journal.pone.0018588

**Published:** 2011-04-05

**Authors:** Ruth Villalonga-Planells, Llorenç Coll-Mulet, Fina Martínez-Soler, Esther Castaño, Juan-Jose Acebes, Pepita Giménez-Bonafé, Joan Gil, Avelina Tortosa

**Affiliations:** 1 Departament de Ciències Fisiològiques II, Institut d'Investigació Biomèdica de Bellvitge-Universitat de Barcelona, L'Hospitalet de Llobregat, Barcelona, Spain; 2 Department of Basic Nursing, Institut d'Investigació Biomèdica de Bellvitge-Universitat de Barcelona, L'Hospitalet de Llobregat, Barcelona, Spain; 3 Serveis Cientificotècnics, Unitat de Biología-Bellvitge, Universitat de Barcelona, L'Hospitalet de Llobregat, Barcelona, Spain; 4 Servei de Neurocirurgia, Institut d'Investigació Biomèdica de Bellvitge-Hospital de Bellvitge, L'Hospitalet de Llobregat, Barcelona, Spain; The University of Chicago, United States of America

## Abstract

Glioblastoma multiforme (GBM) is the most common and aggressive primary brain tumor in adults. Despite concerted efforts to improve current therapies and develop novel clinical approaches, patient survival remains poor. As such, increasing attention has focused on developing new therapeutic strategies that specifically target the apoptotic pathway in order to improve treatment responses. Recently, nutlins, small-molecule antagonists of MDM2, have been developed to inhibit p53-MDM2 interaction and activate p53 signaling in cancer cells. Glioma cell lines and primary cultured glioblastoma cells were treated with nutlin-3a. Nutlin-3a induced p53-dependent G1- and G2-M cell cycle arrest and apoptosis in glioma cell lines with normal *TP53* status. In addition, nutlin-arrested glioma cells show morphological features of senescence and persistent induction of p21 protein. Furthermore, senescence induced by nutlin-3a might be depending on mTOR pathway activity. In wild-type *TP53* primary cultured cells, exposure to nutlin-3a resulted in variable degrees of apoptosis as well as cellular features of senescence. Nutlin-3a-induced apoptosis and senescence were firmly dependent on the presence of functional p53, as revealed by the fact that glioblastoma cells with knockdown p53 with specific siRNA, or cells with mutated or functionally impaired p53 pathway, were completely insensitive to the drug. Finally, we also found that nutlin-3a increased response of glioma cells to radiation therapy. The results provide a basis for the rational use of MDM2 antagonists as a novel treatment option for glioblastoma patients.

## Introduction

The protein p53 is a key regulator of the multiple cellular processes, and depending on the cell type and other factors p53 activation can result in apoptosis, reversible (quiescence) and irreversible cell cycle arrest [1], [2]. p53 is negatively regulated by MDM2 through different mechanisms in coordination with HDMX (MDM4). MDM2 binds the transcription domain of p53 and blocks its ability to activate gene transcription [3], [4]. MDM2 also functions as an E3 ligase, mediating the ubiquitination and proteasome degradation of p53 [4], [5], [6]. In addition, MDM2 can also promote nuclear export of p53 and inhibit its acetylation [7]. Accordingly, MDM2 inhibition could be an effective approach toward enhancing cancer therapy. Nutlins, potent and selective small-molecule antagonists of MDM2, have been shown to activate the p53 pathway in wild-type p53 cell lines of diverse human malignancies both *in vitro* and *in vivo* [8], [9]. Nutlins bind to the p53-binding pocket in the MDM2 protein, thus inhibiting the binding of p53 and activating the p53 pathway in cancer cells with wild-type p53, including solid tumors [10], [11] and hematological malignancies [12], [13], [14], [15]. Nutlin-3a, the active enantiomer of nutlin-3, has been shown to inhibit growth of p53 wild-type human tumors grown as xenografts in nude mice and to induce apoptosis and cell cycle arrest in cancer cell lines that express wild-type p53 [16], [17], [18].

Glioblastoma multiforme (GBM) is the most common and most malignant primary brain tumor in adulthood [19], [20]. Despite treatment efforts including new technological advances in neurosurgery, radiation therapy, and clinical trials with novel therapeutic agents, the vast majority of glioma patients die within 2 years of diagnosis [21], [22], [23]. Different molecular alterations in critical regulatory genes that promote tumor growth, invasion, and resistance to apoptotic stimuli have been identified in human glioblastomas and related to both gliomagenesis and response to therapy [20], [24], [25], [26]. In this sense, the recent TCGA pilot project showed that *TP53* mutations or homozygous deletion and, MDM2 amplification were observed in 35% and 14% of glioblastoma patients, respectively. In addition, amplification of HDMX gene has been observed in only 4% of analyzed samples [25]. As such, increasing attention has focused on developing new therapeutic strategies that specifically target the apoptotic pathway in gliomas in order to improve treatment responses [27].

The purpose of this study is to investigate the antitumor activity of nutlin-3a in glioblastoma cell lines and primary cultured glioblastoma cells. We demonstrate that nutlin-3a induces p53-dependent apoptosis and cellular senescence in wild-type p53 glioma cell lines and primary glioblastoma cultures. Furthermore, we show that nutlin-3a fails to induce apoptosis and cell cycle arrest in glioblastoma cells with mutant p53. Finally, we also found that nutlin-3a enhanced radiation response of glioma cells. Taken together, the results of the present study suggest that MDM2 antagonists may provide a novel treatment option for glioblastoma patients.

## Results

### Nutlin-3a induces cell cycle arrest and apoptosis in wild-type p53 U87MG but not in p53-mutated T98G cells

**Figure 1:**
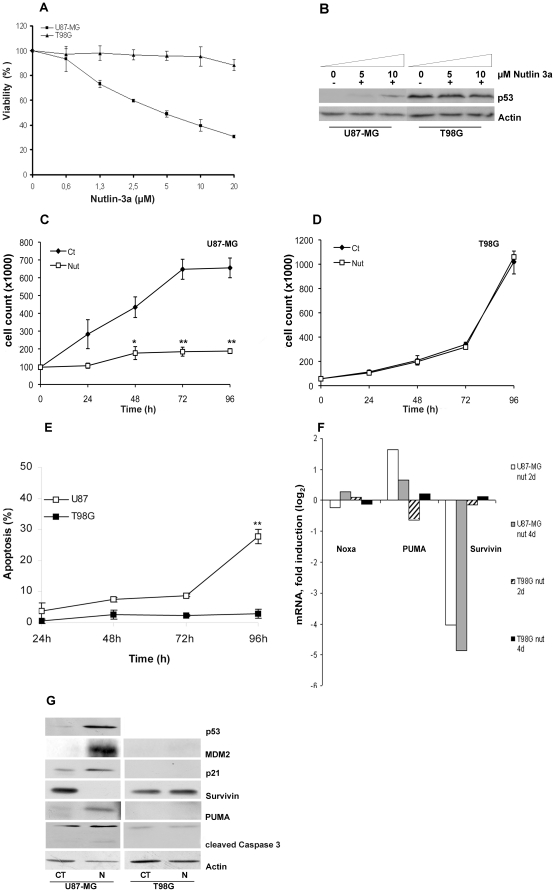
Effect on cell viability and apoptosis of MDM2 antagonists on glioblastoma cell lines. **A**, viability of U87MG (p53 wild-type) and T98G (p53-mutant, negative control) cell lines was assessed by MTT reduction at 96 hours of incubation with either different nutlin-3a concentrations or DMSO treatment (vehicle control). Results are expressed as % compared with DMSO-treated controls and represented as the mean ± standard deviation (sd) for three distinct experiments. U87MG [closed squares] and T98G [closed triangles]. **B**, cells were treated with nutlin-3a (5 or 10 µM) or DMSO (vehicle control) for 24 hours, and then lysed and analyzed by Western blot as described in “Patients, materials and methods”. Treatment with nutlin-3a resulted in a dose-dependent accumulation of p53 protein in U87MG cells whereas no changes were observed in p53-mutant T98G cell line; **C** and **D**, cells were treated with nutlin-3a (10 µM) or DMSO (vehicle control); cells were counted with trypan blue exclusion assay at the indicated days. Points, average of three independent assays expressed as the mean ± sd. (C: U87MG; D: T98G); **E**, time course of nutlin-3a induced apoptosis. U87MG and T98G glioblastoma cell lines, were incubated for 24 to 96 hours with either 10 µM nutlin-3a or DMSO (vehicle control). Apoptosis was measured by surface Annexin V staining and flow cytometry as described in “Patients, materials and methods”. Apoptosis values of treated cells are represented with respect to the DMSO control (vehicle controls). Data are shown as the mean of at least three independent assays ± sd. ** p<0.01, statistical significance (Student's t test); **F**, glioblastoma cell lines U87MG and T98G were treated with 10 µM nutlin-3a or DMSO vehicle for 48 and 96 hours. Expression of apoptosis-related genes was analyzed by RT-MLPA as described in “Patients, materials and methods”. Quantification of mRNA (y axis) is expressed as the base-2 logarithm (log_2_) of the fold induction relative to DMSO-vehicle treated cells. **G**, effect on p53, MDM2, p21, Survivin and PUMA proteins was evaluated 96 hours after nutlin-3a exposure. Cells were treated with nutlin-3a, and then lysed and analyzed by Western blot as described in “Patients, materials and methods”. Immunoblots are representative of at least three independent experiments. CT: DMSO vehicle control; N: 10 µM nutlin-3a.

To determine whether nutlin-3a induced a decrease in cell viability, U87MG (wild-type p53) and T98G (mutant p53, as negative control) human glioblastoma cell lines were evaluated. Both cell lines were incubated either with nutlin-3a at different final concentrations from 0.5 to 20 µM or with DMSO vehicle (untreated control) for 48 h and 96 h, and cell viability was assessed by MTT assay. No significant changes in cell viability were observed in either cell line after 48 hours of nutlin-3a incubation (data not shown). However, as shown in Figure 1A, 96 hours of incubation with nutlin-3a resulted in a dose-dependent reduction in cell viability in the wild-type p53 U87MG cell line whereas no significant decrease in cell viability was observed in mutant-p53 T98G cell line (Figure 1A). In addition, treatment with nutlin-3a resulted in a dose-dependent accumulation of p53 protein in these cells with a maximum at 10 µM (Figure 1B). Indeed, p53-induction did not increase after treatment with nutlin-3a at a final dose of 20 µM (data not shown). Mutant-p53 T98G did not induce p53 after nutlin-3a incubation at different doses (Figure 1B). In light of these results, we carried out subsequent experiments with nutlin-3a at a final dose of 10 µM.

To further investigate the effect of nutlin-3a on cell viability, U87MG and T98G cell lines were incubated either with nutlin-3a at a final concentration of 10 µM or with DMSO vehicle (untreated control), and viable cells were counted with trypan blue exclusion assay at different times from 24 to 96 hours. In U87MG cells, a decrease in the number of viable cells was found 24 hours after incubation when compared to controls. A slight increase in the number of cells was observed 48 hours after treatment; the number of cells then remained stable from 48 to 96 hours (Figure 1C), suggesting that the drug had a cytostatic effect on these cells. No effect on cell counting was observed in T98G mutant-p53 cell line (Figure 1D). To further investigate the effects of nutlin-3 on glioma cells, induction of apoptosis was assessed by annexin V staining positivity. As shown in Figure 1E, U87MG cells showed a time-dependent increase of apoptosis induced by nutlin-3a (from 3.3 to 27% at 24 and 96 hours, respectively) when compared to DMSO vehicle control, whereas no apoptosis was observed in T98G cells, suggesting resistance to MDM2 inhibition in glioblastoma cell lines with mutant p53.

To better characterize the mechanisms by which MDM2 antagonists induced p53-dependent apoptosis, changes in overall apoptosis expression profile by RT-MLPA were analyzed in both glioblastoma cell lines. Upon incubation with nutlin-3a for 48 and 96 hours, U87MG cells showed changes in the mRNA profiles of p53-target genes PUMA and Survivin. Interestingly, an important decrease in the levels of Survivin mRNA was observed together with an accumulation of PUMA mRNA. Another p53-target gene, the pro-apoptotic Bcl-2 family member Noxa, showed no increase in response to nutlin-3a in glioma cell lines (Figure 1F and Figure S1). These data demonstrate the ability of nutlin-3a to activate the intrinsic apoptotic pathway by a p53-dependent mechanism in a glioma cell line that expresses wild-type p53. By contrast, no changes in mRNA profile were observed in the mutant-p53 T98G cell line either at 48 or 96 hours after MDM2 inhibition (Figure 1F and Figure S1).

Results obtained in the mRNA pattern were verified by analysis of protein expression with Western blot. p53, p21, MDM2, PUMA and cleaved caspase 3 proteins increased upon treatment with nutlin-3a in U87MG cells, whereas Survivin protein expression markedly decreased (Figure 1G). T98G cells showed basal mutant p53 protein expression with a slight induction after nutlin-3a exposure. Accordingly, neither reduction of Survivin expression nor induction of p21, MDM2, PUMA or cleaved caspase 3 proteins was observed after MDM2 inhibition in the mutant p53 glioma cell line (Figure 1G).

### Nutlin-3a induces p53-dependent irreversible cell-cycle arrest and senescence in U87 cell line

**Figure 2:**
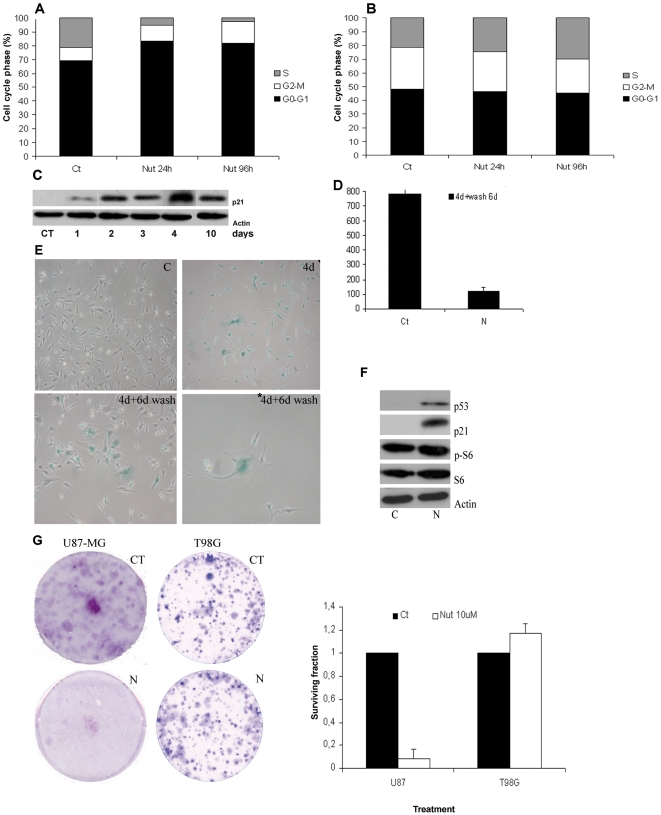
Nutlin-3a induced cell cycle arrest and cellular senescence in U87MG cells. **A** and **B**, glioma cell lines (A: U87MG; B: T98G) were treated either with nutlin-3a or DMSO (vehicle control) for 24 to 96 hours, and cell cycle profile was measured by flow cytometry. Columns, average of three independent determinations; **C**, time-course of p21 protein expression in U87MG cells after nutlin-3a incubation for 1 to 4 days; 10 days showed p21 protein expression on day six after removal of nutlin-3a (day 10 after cells were plated). Immunoblots are representative of at least three independent experiments; **D**, U87MG cells were treated with nutlin-3-a for 4 days, then washed to remove the drug and incubated for additional six days in fresh media. After removal of the drug, cells were trypsinized and counted. Results are shown as mean ± sd. of at least three independent experiments; **E**, representative photomicrographs of U87MG cells stained with SA-βGal after treatment with DMSO vehicle control (Upper left photomicrograph) or with 10 µM nutlin-3a for 4 days (Upper right photomicrograph). Then, the drug was washed out and cells were stained for SA-βGal after incubation for additional 6 days (Lower photomicrograph); **F**, effect on p53, p21, pS6 and S6 proteins was evaluated 48 hours after nutlin-3a exposure by Western blot as described in “Patients, materials and methods”; **G**, representative photomicrographs of colony formation assay. Colonies were counted on day six after nutlin-3-a was removed (day 10 after cells were plated). The number of colonies was scored in three independent experiments and was represented as surviving fraction relative to DMSO vehicle-treated controls ± sd.

Despite weak apoptotic changes observed 24 hours after nutlin-3a incubation, cell growth of p53 wild-type glioblastoma cell line was significantly depressed at that time. To clarify this discordance, the effects of nutlin-3a on cell-cycle distribution were evaluated by flow cytometry at different treatment times, from 24 to 96 hours. Nutlin-3a effectively arrested cell-cycle progression in U87MG cells 24 hours after treatment, depleting the S-phase compartment (from 21% to 3%) and increasing the G0/G1 (from 63% to 80%) and G2/M (from 12% to 17%) phase compartments. Cell-cycle arrest persisted 96 h after nutlin-3a incubation (Figure 2A), suggesting that nutlin-3a might impede cell cycle progression at both the G1/S and G2/M checkpoints in wild-type p53 U87MG cell line. T98G mutant-p53 cells showed no significant differences regarding cell cycle profile when comparing controls (DMSO vehicle) to treated cells (Figure 2B). In addition, nutlin-3a induced p21 expression, an important mediator of p53-dependent cell cycle arrest, 24 h after incubation, and it persisted 96 hours after treatment (Figure 2C).

**Figure 3:**
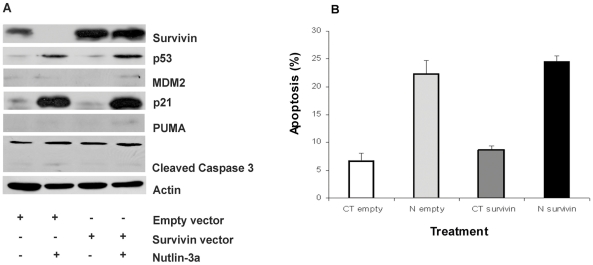
Ectopic overexpression of Survivin fails to suppress nutlin-3a cytotoxicity in wild-type glioma cells. **A**, Immunoblots were performed 48 hours after transfection and 72 hours after treatment either with DMSO or nutlin-3a. Restoration of Survivin did not prevent p53, MDM2, p21, PUMA and cleaved Caspase 3 induction after treatment with nutlin-3a; **B**, Induction of apoptosis in DMSO (CT) and nutlin-3a (N) treated cells 48 hours after transfection either with empty pcDNA3 (empty) or plasmid expression vector pcDNA3-Survivin (survivin) and 72 h after treatment. Apoptosis was measured by surface Annexin V staining and flow cytometry as described in “Patients, materials and methods”. Average of a total of three independent assays ± sd. empty: empty vector; Survivin: Survivin-expressing vector.

To elucidate whether nutlin-3a induces permanent cell cycle arrest and senescence, or reversible cell cycle arrest in glioblastoma cells, the ability of resume proliferation was evaluated. U87MG glioma cell line was incubated with nutlin-3a or DMSO vehicle (untreated control) for 4 days and then the cells were washed to remove nutlin-3a and incubated for additional 6 days in fresh media. After removal of the drug, glioma cells barely resumed proliferation (Figure 2D). We then evaluated whether U87MG treated cells showed features of senescence by staining them with SA-βGal. Nutlin-3a-treated glioma cells acquired an enlarged and flat morphology and expressed the senescence-associated SA-βGal after 4 days of nutlin-3a-incubation (see square 4d in Figure 2E), which persisted upon removal the drug (see square 4d+wash 6d in Figure 2E). Furthermore, persistent induction of p21 protein was also observed, thus suggesting that cell-cycle arrest exceeded the duration of treatment (Figure 3C). Additionally, irreversible cell cycle arrest by nutlin-3a was also confirmed by colony forming assay. U87MG cells presented a significant decrease in the capacity to form colonies upon removal of nutlin-3a when compared to control (Figure 2G). Indeed, most of the remaining cells exhibited a large/flat morphology and were SA-βGal-positive (see square *4d+wash 6d in Figure 2E). Induction of p53 can cause apoptosis, reversible cell cycle arrest (quiescence), and irreversible cell cycle arrest (cellular senescence). In addition, recent studies have demonstrated that when the cell cycle is blocked, activation of mTOR is required for induction of senescence. Thus, in spite of its ability to induce cell cycle arrest, p53 can act as a suppressor of cellular senescence when inhibits mTOR pathway [28], [29], [30], [31], [32], [33]. To further evaluate whether senescence induced by nutlin-3a in glioma cells is depending on mTOR signaling, we evaluated activity of mTOR pathway after treatment. Western blot analysis of S6 phosphorylation protein suggested that mTOR pathway remains active after nutlin-3a in glioma cells (Figure 2F). Taken together, these results confirm that nutlin-3a induces senescence in U87MG cells and suggest that it might be dependent on mTOR pathway activity.

### Silencing of p53, but not overexpression of Survivin, suppresses nutlin-3a cytotoxicity in wild-type p53 U87 cell line

Owing to the potential antiapoptotic role of Survivin, we asked whether overexpression of exogenous Survivin would protect glioma cells from nutlin-induced apoptosis. Western analysis showed substantial elevation of the basal Survivin levels in the cells transfected with Survivin expression construct compared with the vector controls (Figure 3A). Unexpectedly, restoration of Survivin did not prevent p53, p21, MDM2, Puma and cleaved caspase 3 protein induction after nutlin-3a incubation (Figure 3A). Furthermore, ectopic overexpression of Survivin and treatment with nutlin-3a resulted in a non-significant reduction of apoptosis induction (6.8% in pcDNA-empty and 8.55% in pcDNA-Survivin-transfected DMSO control cells, and 21.2% in pcDNA-empty and 24.6% in pcDNA-Survivin-transfected nutlin-3a treated cells) (Figure 3B). No changes in cell cycle profile were observed between pcDNA-empty and pcDNA-Survivin transfected cells after nutlin-3a incubation (data not shown).

To investigate whether knocking down p53 rendered U87MG cells resistant to nutlin-3a apoptosis and cell-cycle arrest induction, cells were transfected with p53 siRNA (or control siRNA) and treated with nutlin-3a as described above. Immunoblotting of U87MG cells demonstrated that p53, p21 and MDM2 were induced after 24, 48 and 72 hours of nutlin-3a treatment or after transfection with control siRNA, whereas they decreased to near baseline levels after p53 siRNA. In addition, MDM2 antagonists and p53 siRNA resulted in reduced PUMA and cleaved caspase-3 induction when compared with cells transfected with control siRNA (Figure S2A). As expected, p53 siRNA followed by nutlin-3a treatment resulted in maintenance of cell viability (Figure S2B) and reduced apoptosis induction when compared with control siRNA (Figure S2C).

### Nutlin-3a induces p53-dependent apoptosis, cell cycle arrest and senescence in p53 wild-type primary cultured glioblastoma cells

**Figure 4:**
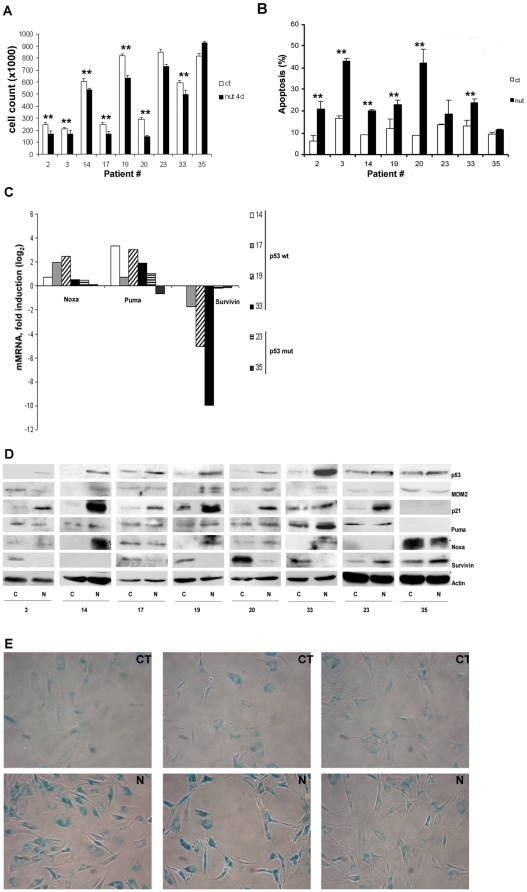
Effects of MDM2 antagonists on primary cultured glioblastoma cells. **A**, cell count on nine primary cultured glioblastoma cells treated with DMSO (vehicle control) (□) or treated with 10 µM nutlin 3-a (▪); *Columns*, average of two independent assays; bars sd. ** p<0.01, statistical significance of nutlin-3a treated cells with respect to the untreated controls (Student's t test). **B**, effect of nutlin-3a on apoptosis induction in eight primary cultured glioblastoma cells treated with DMSO (vehicle control) (□) or treated with 10 µM nutlin 3-a (▪); *Columns*, average of two independent assays; Apoptosis was measured by surface Annexin V staining and flow cytometry as described in “Patients, materials and methods”. *Columns*, average of two independent assays; bars sd. ** p<0.01, statistical significance of nutlin-3a treated cells with respect to the untreated controls (Student's t test); **C**, expression of p53 target genes was analyzed by RT-MLPA as described in “Patients, materials and methods”. The results are shown as logarithmic fold induction relative to untreated cells; **D**, effect on p53, MDM2, p21, Puma, Noxa and Survivin was evaluated 48 hours after nutlin-3a exposure by Western blot. Cells were treated with nutlin-3a, and then lysed and analyzed by Western blot as described in “Patients, materials and methods” (CT: DMSO vehicle control; N: 10 µM nutlin-3a); **E** representative photomicrographs of three glioblastoma cultured cells stained with SA-βGal after treatment with 10 µM nutlin-3a (N) or with DMSO vehicle control (C) for 4 days.

**Figure 5:**
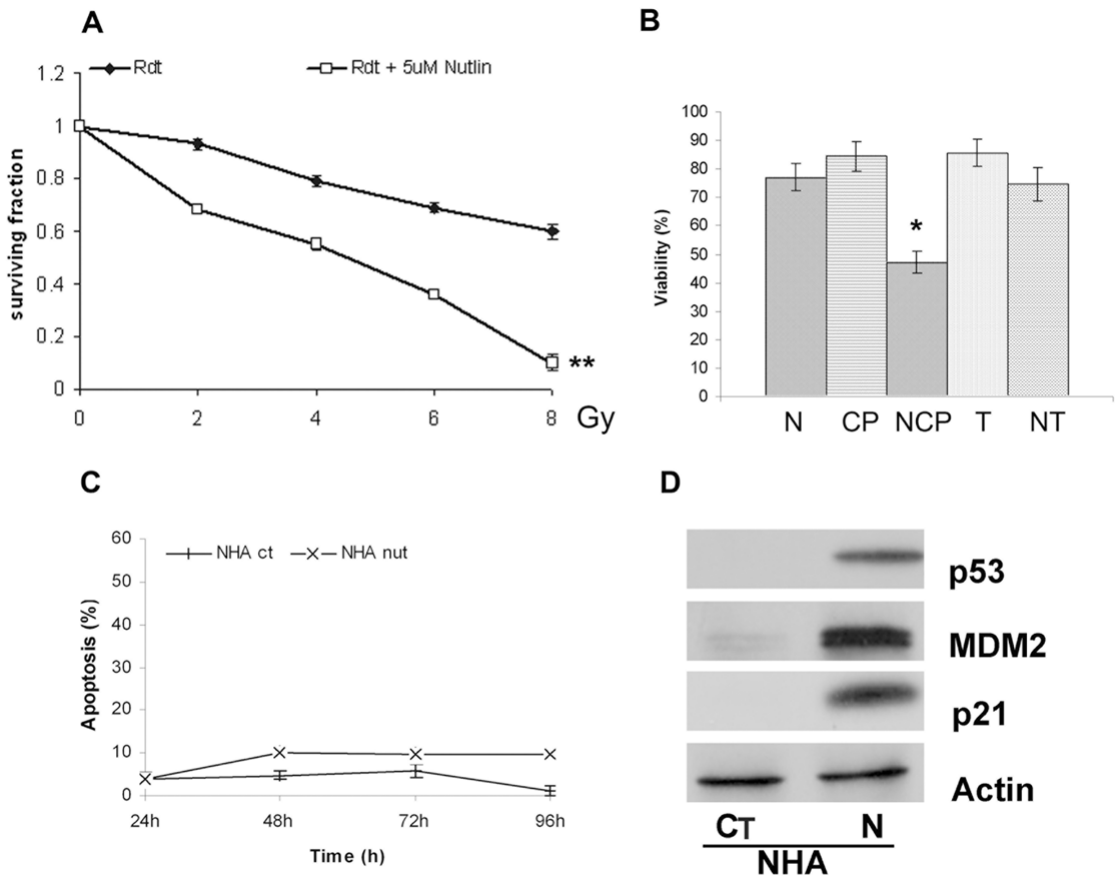
Nutlin-3a enhances radiation response in wild-type p53 glioma cells. **A**, clonogenic assays of p53-wild-type U87MG cells after 2 h pre-treatment with DMSO (vehicle control) (♦) or nutlin-3a (0.5 µM) (□)followed by exposure to X-ray irradiation at 0, 2, 4 6 or 8Gy; The number of colonies were represented as surviving fraction. Data are shown as the mean of at least three independent experiments; **B**, effect on cell viability assessed by MTT assay of p53-wild-type U87MG cells after being treated with nutlin-3a 0.5 µM (N), 2 µM cisplatin (CP) and both (NCP); or being treated with 1 µM temozolomide (T) and both (NT). Data are shown as the mean of at least three independent experiments ± sd. * p<0.05, ** p<0.0001, statistical significance (Student's t test); C, time course of nutlin-3a induced apoptosis. normal human astrocytes (NHA), were incubated for 24 to 96 hours with either 10 µM nutlin-3a or DMSO (vehicle control). Apoptosis was measured by surface Annexin V staining and flow cytometry as described in “Patients, materials and methods”.). Data are shown as the mean of at least three independent assays ± sd. ** p<0.01, statistical significance (Student's t test); D, effect on p53, MDM2, and p21 proteins of nutlin-3a incubation in NHA. Cells were treated with nutlin-3a, and then lysed and analyzed by Western blot as described in “Patients, materials and methods” (CT: DMSO treated vehicle control; N: 10 µM nutlin-3a).

To further analyze the ability of nutlin-3a to induce apoptosis in glioblastoma, primary cultured glioblastoma cells obtained from patients and grown in culture for a very short period of time were used. Primary cultured glioblastoma cells from nine different patients were incubated with nutlin-3a for 48 and 96 hours. Short-time incubation with MDM2 inhibitor did not demonstrate changes in the number of cells between control and treated cultures (data not shown). As shown in Figure 4A, a significant decrease (Student t test, p = 0.005) in the number of cells was observed in all but two patients (#23 and #35) treated with nutlin-3a when compared to controls. To further evaluate nutlin-3a's effect, induction of apoptosis by surface annexin V positivity was analyzed in all but one patient (#17, due to a lack of cells). Cell viability decreased from 91%±2% to 78%±10% (Student t test, p = 0.012) after incubation with nutlin-3a for 96 hours in wild-type p53 primary culture. There were important case-to-case differences in apoptosis induction after nutlin-3a incubation (Figure 5B). However, in all glioblastoma patients with wild-type p53 (Table 1), nutlin-3a had pro-apoptotic and anti-proliferative effects (Figure 4A and B). Two patients (#23 and #35) presented a very low non-significant apoptosis induction after MDM2 inhibition when compared to controls (Figure 4B). In addition, one patient with no apoptosis induction upon nutlin-3a incubation had a p53-mutation (R306X in patient #23), and patient #35 carried a *TP53* polymorphism (R72P). Variant p53-P72 has previously been described as having a weaker apoptotic potential in lung cancer cells. However, it is not yet known how universal these functional differences between the two variants might be in different cell types, nor whether they are relevant *in vivo* [34], [35]. In addition, response to MDM2-inhibitors was independent of MGMT promoter methylation status (Table 1). Together, the present results confirm the hypothesis that inhibition of MDM2-p53 binding in primary cultured glioblastoma cells with functional p53 pathway induces apoptosis. Additionally, primary cultured glioblastoma cells with mutant p53 are resistant to nutlin-3a apoptosis induction.

**Table 1:** Patient characteristics.

Patient id.	Age	Diagnosis	W.H.O. grade	p 53 status	MGMT promoter methylation status	EGFRvIII mutation
02	65	GBM	IV	Wt	Unmethylated	+
03	74	GBM	IV	Wt	Methylated	Nd
14	49	GBM	IV	Wt	Unmethylated	Nd
17	65	GBM	IV	Wt	Nd	Nd
19	77	GBM	IV	Wt	Methylated	Nd
20	76	GBM	IV	Wt	Unmethylated	-
23	72	GBM	IV	Mutated	Nd	-
33	69	GBM	IV	Wt	Unmethylated	-
35	45	GBM	IV	P	Methylated	+

GBM: glioblastoma multiforme; Wt: wild-type; P: Polymorphism R72P; Nd: not done.

To further analyze p53-dependent apoptosis in primary cultured glioblastoma cells, changes in apoptosis-related gene expression profile were evaluated by RT-MLPA in six samples. Three patients were excluded from the analysis due to low RNA yield. Nutlin-3a treatment for 48 and 96 hours induced changes in *PUMA, Noxa and Survivin* gene expression in wild-type p53 samples. The most noteworthy result was the decrease in Survivin mRNA expression observed in these patients with a reduction of cell viability and increased apoptosis after nutlin-3a treatment (patients #14, 17, 19 and 33) (Figure 4C and Figure S3). An increase in PUMA mRNA expression was found in all but one patient (patients #14, 19 and 33). In addition, an increase in Noxa mRNA expression was also observed in patients #14 and #17.

To verify changes observed in the pro-apoptotic mRNA expression profile, primary cultured glioblastoma cells were analyzed by Western blot for expression levels of p53, MDM2, p21, PUMA, Noxa and Survivin proteins (Figure 4D). p53 and p21 protein accumulation was observed in primary cultured glioblastoma cells with wild-type p53 after MDM2 inhibition. Regarding MDM2, all patients with p53 wild-type resulted in nutlin-3a induced protein expression (Figure 4D). Expression of baseline MDM2 protein was observed in 3 out of 6 patients with wild-type p53 (patients #3, 17 and 20) which resulted in higher nutlin-3a apoptosis induction (Figures 4B and 4D). A decrease in Survivin protein expression was observed in all patients with nutlin-3a-induced apoptosis, consistent with the low levels of Survivin mRNA previously found at RT-MLPA. Regarding Noxa protein expression, 5 out of 6 patients with p53 wild-type demonstrated induction after MDM2 inhibition (patients #3, 14, 19, 20 and 33), consistent with results found at the mRNA level. Finally, an increase in PUMA protein expression was observed in 4 out of 6 patients with wild-type p53 after nutlin-3a incubation (patients #14, 19, 20 and 33). In this case, protein results were also consistent with those found at the mRNA level (Figure 4C and 4D). No changes in Survivin, PUMA or Noxa mRNA expression were observed in p53-mutant patients #23 and #35, resistant to MDM2 inhibition (Figure 4C and Figure S3). At the protein level, both patients showed high baseline expression of p53 protein without induction after drug treatment. An increase of Survivin protein expression was also observed upon MDM2 inhibition. In addition, patient #23 exhibited an increase in p21 protein expression after nutlin-3a treatment. Changes in other p53 target proteins were not observed (Figure 4D). Patient #35, carrying a p53 polymorphism [34] that could partially explain the lack of apoptosis induction, exhibited resistance to nutlin-3a treatment.

To evaluate whether nutlin-3a induced senescence in wild-type p53 primary glioblastoma cultures, cells were treated with either nutlin-3a or DMSO (vehicle control), and the expression of senescence marker SA-βGAL was evaluated 4 days after incubation. Upon nutlin-3a incubation the percentage of positive cells increased when compared to DMSO vehicle, from 28%±9% to 82%±8%, respectively (Student t test, p<0.001) (Figure 4E).

### Nutlin-3a enhances radiation response in wild-type p53 glioma cells

It has been shown previously that cells in G1 are approximately twice as radiosensitive as cells in S-phase [36], and therefore we considered the possibility that the cell cycle arrest that we observed in wild-type glioma cells might also lead to increased radiosensitivity. Thus, we performed *in vitro* clonogenic assays for glioma cells after 2 h pre-treatment with nutlin-3a (0.5 *µ*M) or DMSO (vehicle control) followed by exposure to X-ray irradiation at 0, 2, 4 6 or 8Gy (Clinac 600 CD, M/S Varian AG, USA). Pre-treatment with nutlin-3a had a significant effect on the p53 wild-type cells, making them significantly more radiosensitive (comparison of surviving fraction values; *P*<0.0001, log-rank scale) (Figure 5A). As expected from our earlier data, nutlin-3a treatment had no significant effect on mutant-p53 glioma cells. In addition, incubation of U87MG cells with nutlin-3a (0.5 µM) in combination with cisplatin (2 µM) significantly decreased cell viability when compared with each treatment alone (Student t test, p<0.05) with a combined index of 0.16, suggestive of synergistic effect (Figure 5B). No effect was observed when we combined nutlin-3a with temozolomide at different doses.

Finally, we also evaluated the effect of nutlin3-a on normal brain cells. Normal human astrocytes (NHA) were incubated with nutlin-3a at a final concentration of 10 µM and we observed a decrease in cell viability in MTT assay (100% cell viability in DMSO vehicle and 40% in nutlin-3a after 96 h of incubation). A slight induction of apoptosis was also observed in NHA with non-significant differences when compared with mutant-p53 T98G cell line (negative control) (Figure 5C). In addition, western-blot analyses demonstrate an increase in p53, MDM2 and p21 protein expression (Figure 5D). The discordance observed when comparing the decrease in cell number assessed by MTT and apoptosis induction together with p21 induction strongly suggests that nutlin-3a mainly induced cell cycle arrest but not apoptosis in NHA. These results agree with previous data reported that MDM2 antagonists induce reversible cell cycle arrest but not cell death in normal cells [8] and indicate that wild-type-p53 glioblastoma cells are more sensitive to nutlin-3a treatment than NHA.

## Discussion

In this study, we show that nutlin-3a effectively stabilizes and activates p53 in glioblastoma cell lines as well as in primary glioblastoma cultured cells with wild-type *TP53*, leading to induction of p53-dependent cell-cycle arrest and apoptosis. In addition, our results also indicate that in wild-type p53 glioblastoma cells, cell cycle arrest is the primary response to MDM2 inhibition, whereas induction of apoptosis varies substantially. In fact, these findings are in line with previous reports in which the antitumor activity of nutlin-3a was demonstrated in a variety of wild-type p53 cancer cells of both solid tumors [10], [11], [15] and hematological malignancies [12], [13], [14]. Overall, the most common effect in solid-tumors is cell cycle arrest. Nutlin-induced apoptosis is more likely a reflection of a cell's ability to undergo p53-dependent apoptosis [17], [18]. Indeed, cancer cell lines can differ significantly in their apoptotic response to similar levels of p53 activation. Accordingly, the results of the present study suggest that in glioblastoma cells nutlin-3a primarily induces p53-dependent cell-cycle arrest and senescence, and to a lesser extent, apoptosis.

Our study provides direct evidence that both cell cycle arrest and senescence, and apoptosis induced by nutlin-3a are due to activation of the p53 pathway. p53 promotes G1 cell arrest through its ability to induce p21 expression [37], and p53 and p21 levels are increased by nutlin-3a treatment in wild-type p53 glioblastoma cells. In addition, our experiments show that knocking down p53 expression using siRNA restores cell viability and decreases p21 to normal levels. Moreover, p53 knockdown also diminishes apoptosis, and proapoptotic PUMA and cleaved caspase 3 protein induction, thus demonstrating that the nutlin-induced cell cycle arrest and apoptosis observed in glioblastoma cells are p53-dependent. The fact that p53-mutant T98G cells and glioblastoma primary cultures are insensitive to nutlin-3a further reinforces the above-mentioned concept.

Interestingly, our results show that MDM2 inhibition induces cellular senescence in p53 wild-type glioblastoma cells. Thus, wild-type p53 glioblastoma cells barely resume their proliferative potential and almost completely lost their capacity to form colonies, acquiring an enlarged and flat morphology with SA-βGal-positive expression upon nutlin-3a removal. Senescence induction by nutlin-3a has been previously reported in fibroblasts and fibrosarcoma cells [28], T-cell leukemia cells [29] and neuroblastoma [30]; however, to our knowledge, this is the first time to report that nutlin-3a induces senescence in glioma cells. Although p53 has been regarded as a canonical inducer of senescence [2], recent studies have found that p53 could also negatively regulate senescence. Mechanism undergone senescence-induced by nutlin-3a may be dependent on both p53 induction of cell cycle arrest and p53 regulation of mTOR pathway. Indeed, the principal role of p53 is to promote cell cycle arrest but the subsequent fate of the arrested cells may be dependent on its effect on mTOR signaling [29], [30], [31], [32], [33]. According to the above-mentioned mechanisms, our results suggest that nutlin-3a induces senescence in p53 wild-type glioblastoma cells by its failure to inhibit the mTOR pathway.

In the present study, nutlin-3a was found to down-regulate Survivin in glioma cell lines and primary glioblastoma cells with wild-type p53. Survivin, a member of the inhibitor of apoptosis protein (IAP) family, functions as a key regulator of mitosis and programmed cell death. Survivin, in cooperation with other IAP molecules (such as hepatitis B X-interacting and X-linked IAP proteins), selectively blocks apoptosis at the level of effector caspases [38], [39]. Survivin gene expression is transcriptionally repressed by wild-type p53, and down-regulation of Survivin has been reported to induce a block of cell entry into S phase and cell-cycle arrest in G2/M phase^35^. Uchida et al.[40] reported that siRNA against Survivin was able to induce apoptosis and suppress cell growth both *in vitro* and *in vivo* in U251 glioma cells. Surprisingly, in our study, ectopic overexpression of Survivin failed to rescue U87MG cells from the cytotoxic effects of MDM2 inhibition. Previous studies have demonstrated that ectopic overexpression of Survivin resulted in reduction of cell death in U87MG cells upon TRAIL-quercetin incubation[41]. The results of our study suggest that upon nutlin-3a incubation, down-regulation of Survivin might be redundant with other induced changes, and its overexpression is unable by itself to abolish cell cycle arrest and apoptosis in U87MG cells.

Finally, we found that nutlin-3a enhanced radiation response of wild-type p53 glioma cell lines. Pre-treatment with nutlin-3a induces cell cycle arrest making glioma cells more sensitive to radiation therapy. Further studies are needed to better elucidate the mechanisms underling above presented data. These results along with the demonstration of pleiotropic activities of nutlin-3a by inducing cell cycle arrest and senescence, and apoptosis in glioblastoma cells make MDM2 inhibitors particularly attractive for the treatment of glioblastoma patients.

## Materials and Methods

### Ethics Statement

Written informed consent was obtained from all patients in accordance with the *Hospital de Bellvitge* Ethics Committee. The study was approved by the *Hospital de Bellvitge* Ethics Committee.

### Cell lines and primary cultured glioblastoma cells

U87MG (*TP53* wild-type) and T98G (*TP53* mutant, M237I) glioma cell lines were obtained from the American Type Culture Collection and cultured with DMEM with 10% fetal calf serum (FCS) supplemented with L-glutamine (2 mM) and penicillin-streptomycin (100 U/ml-100 µg/ml). Normal human astrocytes (NHA) were obtained from Lonza (Lonza Cologne AG, Germany) and were cultured with astrocyte basal medium with 10% fetal bovine serum supplemented with 0.1% gentamycin sulfate-amphotericine B, 0.1% ascorbic acid, 0.25% insulin, 1% L-glutamine and 0.1% hEGF. For primary cultured glioblastoma cells, tumor samples were obtained from nine patients undergoing surgical treatment for a glioblastoma multiforme (W.H.O grade IV [20]) at the neurosurgical department of the *Hospital de Bellvitge*, and these were processed within 1 h after resection. Table 1 provides details of patients whose tumors were assayed in this study. The tumor fragment was rinsed with Hank's Balanced Salt Solution (HBSS) and mechanically dissociated into pieces of ∼1–5 mm^3^. Tissue minced pieces were incubated with collagenase I at a final concentration of 200 U/ml in HBSS for 1 hr at 37°C with constant vigorous agitation. The cellular suspension was then washed with DMEM supplemented with 10% heat-inactivated FCS, 2 mM glutamine, and 100 U penicillin and 100 µg/ml streptomycin (culture growth medium). After centrifugation, a thin red strip at the top of the cell pellet corresponding to the remaining erythrocytes was delicately discarded and the pellet was resuspended in culture growth medium. The cellular suspension was plated in 60 mm tissue culture plates and maintained at 37°C in a humidified atmosphere containing 5% carbon dioxide. Cell cultures were subsequently split 1∶2 when confluent and experiments were done before pasages 3–5. All experiments performed with primary cultured glioblastoma cells were repeated at least twice in triplicate.

### Cell viability analysis

The viability of cultured cells was determined by assaying the reduction of MTT [3-[4,5-dimethylthazol-2-yl]-2,5-diphenyl tetrazolium-Bromide] (Sigma Chemical Co.) to formazan. Glioma cell lines were plated in a 96-well plate, 5000 cells/well in a final volume of 100 µl. Cultures were treated with DMSO vehicle (untreated control) or increasing concentrations of nutlin-3a (0.5–20 µM, kindly provided by Dr. L. Vassilev, Hoffmann-La Roche) and evaluated at different times from 24 to 96 hours. After the indicated treatment, cells were incubated for 2 h at 37°C in DMEM containing 10 µM MTT (diluted in PBS). The blue MTT formazan precipitate was then dissolved in 100 µl of DMSO, and the absorbance was measured at 540 nm on a multiwell plate reader.

Cell viability was also assessed by counting the adherent cells with or without treatment (DMSO vehicle control). Glioma cell lines and primary cultured glioblastoma cells were seeded in culture growth medium and incubated with nutlin-3a or DMSO vehicle (untreated control). Experiments were performed at ∼70% cell confluence. For cell counting, attached cells were trypsinized at different times from 24 to 96 hours after treatment. Cell counting was determined by trypan blue exclusion assay in a Neubauer chamber slide.

### Analysis of apoptosis by flow cytometry

Apoptosis was measured by surface Annexin V staining and flow cytometry. Treated and untreated cells were washed in phosphate-buffered saline (PBS), trypsinized and incubated with Annexin V–FITC (Bender MedSystems, Burlingame, CA) for 10 minutes. Cells were then diluted with Annexin-binding buffer to a volume of 400 µL with propidium iodide. Samples were analyzed with FACSCalibur and BD CellQuest Pro software (Becton Dickinson, Mountain View, CA).

### Cell cycle

After treatment, both floating and attached cells were fixed with 70% ethanol, resuspended in PBS/1%FBS, and treated with RNase A. Propidium iodide was added to the cells and samples were analyzed by flow cytometry in a FACSCalibur (Becton Dickinson, Mountain View, CA). Cell-cycle profile analysis of the DNA histograms of integrated red fluorescence was performed with Modfit LT software (Verity Sotware, Inc, Topsham, ME).

### RT-MLPA

RNA was analyzed by reverse transcriptase multiplex ligation-dependent probe amplification (RT-MLPA) using SALSA MLPA KIT R011 Apoptosis mRNA from MRC-Holland (Amsterdam, The Netherlands) for the simultaneous detection of 38 messenger RNA molecules [42]. Briefly, RNA samples (200 ng total RNA) were first reverse-transcribed using a gene-specific probe mix. After adding the probe mix, the resulting cDNA was allowed to hybridize overnight at 60°C. After ligation of both probe pairs and inactivation of ligase, PCR was performed in a volume of 50 µl containing 10 µl of the ligation reaction mixture with one unlabeled and one FAM labeled primer (33 cycles, 30 seconds at 95°C; 30 seconds at 60°C, and 1 minute at 72°C). Fragments were separated by capillary electrophoresis on a 48-capillary ABI-Prism 3730 Genetic Analyzer (Applied Biosystems/Hitachi, Foster City, CA). Peak area and height were measured using GeneScan analysis software (Applied Biosystems). The levels of mRNA for each gene were expressed as a normalized ratio of the peak area divided by the peak area of a control gene, resulting in the relative abundance of mRNAs in the genes of interest. The probe set contains probes for mRNAs of 30 apoptosis-related proteins. Areas were normalized to β2-Microglobuline.

### Western blot analysis

Cells were lysed with Laemmli sample buffer, and equal amounts of protein (50 µg) estimated by the BCA Protein Assay (Pierce, Rockford, IL) were separated by electrophoresis on 8% or 12% polyacrylamide gel and transferred to a PDVF membrane (Amersham, Buckinghamshire, UK). After blocking for 1 hour with 5% dried skimmed milk in TBST, the blots were incubated overnight at 4°C with the specific primary antibody. Primary antibodies used were p53 (Neomarkers, Fremont, CA), p21 (Santa Cruz Biotechnology, Santa Cruz, CA), MDM2, PUMA and Noxa (Abcam, Cambridge, UK), Survivin (Novus Biologicals, Littleton, CO), and S6, pS6 and cleaved Caspase 3 (Cell Signaling Technology, Beverly, MA). Membranes were subsequently incubated with a secondary antibody conjugated to horseradish peroxidase and developed using a chemiluminescent (ECL) detection system (Amersham, Buckinghamshire, UK). Bands were analyzed by densitometry and the numerical data were subjected to t test.

### Detection of *TP53* mutations

DNA was obtained using the AllPrep DNA/RNA Mini Kit (Quiagen), and quantified with NanoDrop ND-1000 (Thermo Fisher Scientific/ Waltham, MA). For the PCR reaction, specific primers obtained from IARC TP53 Mutation Database website (http://www-p53.iarc.fr) were used for each exon (exons 2 to 10). Samples were purified with EZNA Cycle-Pure Kit (Omega Biotek) and subsequently sequenced with big dye 3.1 PCR reaction, and analyzed by means of capillary electrophoresis (Applied Biosystem/Hitachi, Foster City, CA, USA). Finally, sequences were evaluated with Gen Tool 1.0 (Biotools/Canada) and Multalin 5.4.1 software (http://www-archbac.u-psud.fr).

### Methylation-specific PCR

Genomic DNA was isolated from frozen tumor using the AllPrep DNA/RNA Mini Kit (Quiagen). DNA methylation status of CpG islands at the enzyme O^6^-methylguanine methyltransferase (MGMT) promoter was determined by methylation-specific PCR (MSP), as previously described [43], with some modifications.

### Senescence associated β-galactosidase staining

Cells (5×10^4^) were plated in 6.0-cm-diameter plates and treated with 10 µM nutlin-3a or DMSO (vehicle control). After for 4 days, cells were washed to remove nutlin-3a and incubated for additional 6 days in fresh media. Senescence β-galactosidase (SA- βGal) staining was performed using the Senescence-βGal Staining Kit (Cell Signaling Technology, Beverly, MA) following the manufacturer's instructions. Cells were considered positive when the cytoplasm was stained with SA- βGal.

### Colony formation assays

To assess clonogenic ability, cells were plated in six-well plates in appropriate dilutions and treated with nutlin-3a or DMSO (vehicle control). After for 4 days, cells were washed to remove nutlin-3a and incubated for additional 6 days in fresh media. Then, colonies were fixed with 2% ethanol and stained with 0.5% crystal violet. After staining, colonies were counted with a cutoff of 50 viable cells, and plating efficiency of cells was determined by calculating the ratio of the number of colonies formed to the number of cells seeded in DMSO-treated vehicle controls. Surviving fraction after treatment was calculated, with plating efficiency taken into consideration, according to a previous published protocol [44]. Each set of experiments was done in triplicate.

### Transfection and RNA interfering assays

U87MG cells were transiently transfected either with empty pcDNA3 (negative control) or plasmid expression vector pcDNA3-Survivin (kindly provided by Dr. D.C. Altieri, New Haven, Connecticut) along with pcDNA-GFP using Amaxa Nucleofector device (protocol U029; Amaxa Biosystems, Lonza). Transfection efficiency was calculated by fluorescence microscopy and was always above the 80%. Forty-eight hours after transfection, cells were treated either with 10 µM nutlin-3a or DMSO vehicle (untreated control) and evaluated for cell viability, apoptosis induction and protein expression at 48 and 72 hours after treatment.

U87MG cells were suspended in 100 µL of solution T (Amaxa) and nucleofected either with 300 nmol/L of non-targeting negative-control human small interference RNA (Negative Universal Control siRNA, Invitrogen) or 300 nmol/L human p53 siRNA (TP53 UHS40367 siRNA, Invitrogen) with an Amaxa nucleofector device (protocol X001; Amaxa Biosystems, Lonza). Six hours after transfection, cells were treated either with 10 µM nutlin-3a or DMSO vehicle (untreated control), and evaluated for cell viability, apoptosis induction and protein expression at 48 and 72 hours after treatment.

### Statistical analysis

Results are expressed as mean ± standard deviation (SD) of values obtained in at least three independent experiments. Differences between samples were analyzed with Student t test. Differences reaching a p value of 0.05 were considered significant. All calculations were performed using the 14.0 SPSS software package (SPSS Inc., Chicago, IL). The combination index (CI) was calculated for a 2-drug combination using Biosoft CalculSyn program (Fergurson, MO). A CI of 1 indicates an additive effect; a CI above 1 an antagonistic effect; and a CI below 1, a synergistic effect.

## Supporting Information

Figure S1 Glioblastoma cell lines U87MG and T98G were treated with 10 µM nutlin-3a for 48 hours and 96 hours. Expression of apoptosis-related genes was analyzed by RT-MLPA as described in “Patients, materials and methods”. The results are shown as fold induction relative to untreated cells.
(TIF)

Figure S2 Silencing of p53 suppresses nutlin-3a cytotoxicity in wild-type p53 U87 cells. A, immunoblots demonstrating silencing of p53 protein in U87MG cells 48 hours after transfection and 72 hours after nutlin-3a treatment. Knockdown of p53 prevent U87MG cells to p53, p21, MDM2, Puma and cleaved caspase 3 proteins induction as well as Survivin down-regulation. Immunoblots are representative of at least three independent experiments. B, U87MG cells transfected either with p53 specific siRNA or negative-control siRNA and 6 hours later treated with nutlin-3a (10 µM) or DMSO (vehicle control; ct). The number of viable cells was counted with trypan blue exclusion assay at 48 and 72 hours. Points, average of three independent assays expressed as the mean ± sd. C, time course of nutlin-3a induced apoptosis in U87MG cells 6 hours after transfection and 48 and 72 h after treatment. Apoptosis was measured by surface Annexin V staining and flow cytometry as described in “Patients, materials and methods”. Average of a total of three independent assays ± sd.
(TIF)

Figure S3 Primary cultured glioblastoma cells were treated with 10 µM nutlin-3a for 96 hours. Expression of apoptosis-related genes was analyzed by RT-MLPA as described in “Patients, materials and methods”. The results are shown as fold induction relative to untreated cells.
(TIF)
