# Supplementary figures and images for: Activation of p53 by Nutlin-3a Induces Apoptosis and Cellular Senescence in Human Glioblastoma Multiforme
Source: PLoS One. 2011 Apr 5;6(4):e18588. doi: 10.1371/journal.pone.0018588 (PMC3071734; doi:10.1371/journal.pone.0018588)

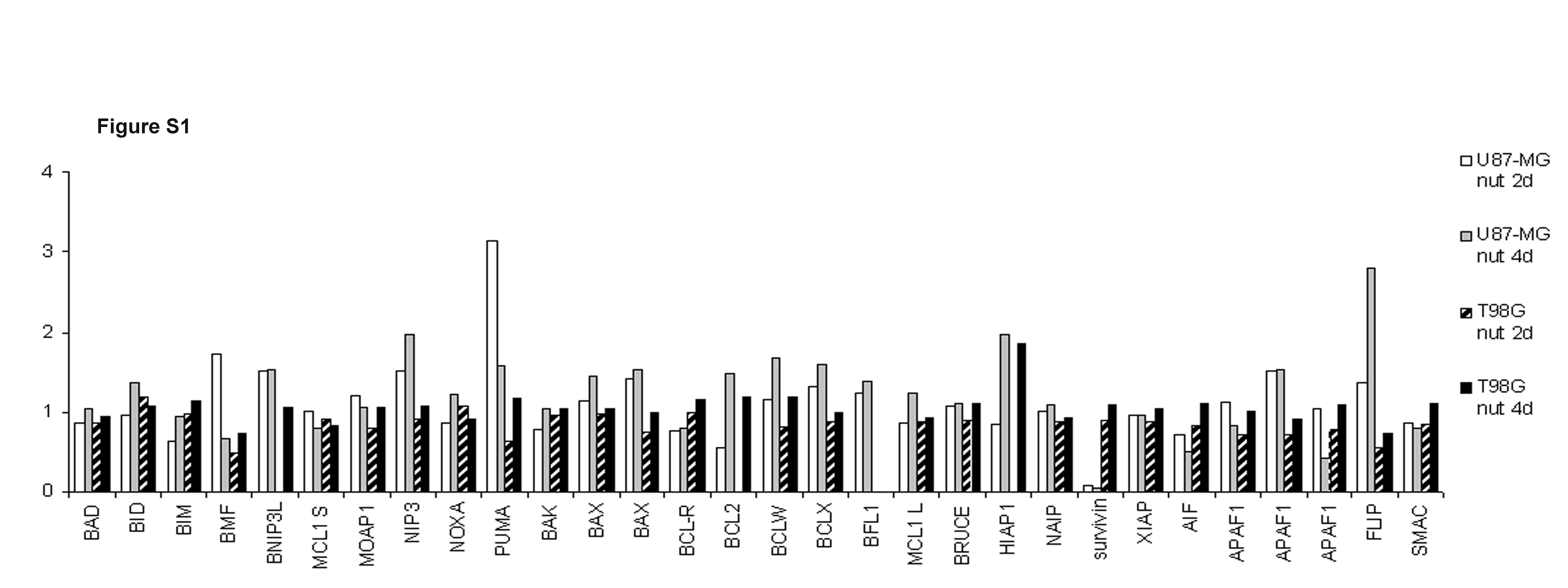

Supplement: Figure S1 — Glioblastoma cell lines U87MG and T98G were treated with 10 µM nutlin-3a for 48 hours and 96 hours. Expression of apoptosis-related genes was analyzed by RT-MLPA as described in “Patients, materials and methods”. The results are shown as fold induction relative to untreated cells. (TIF) [file pone.0018588.s001.tif]

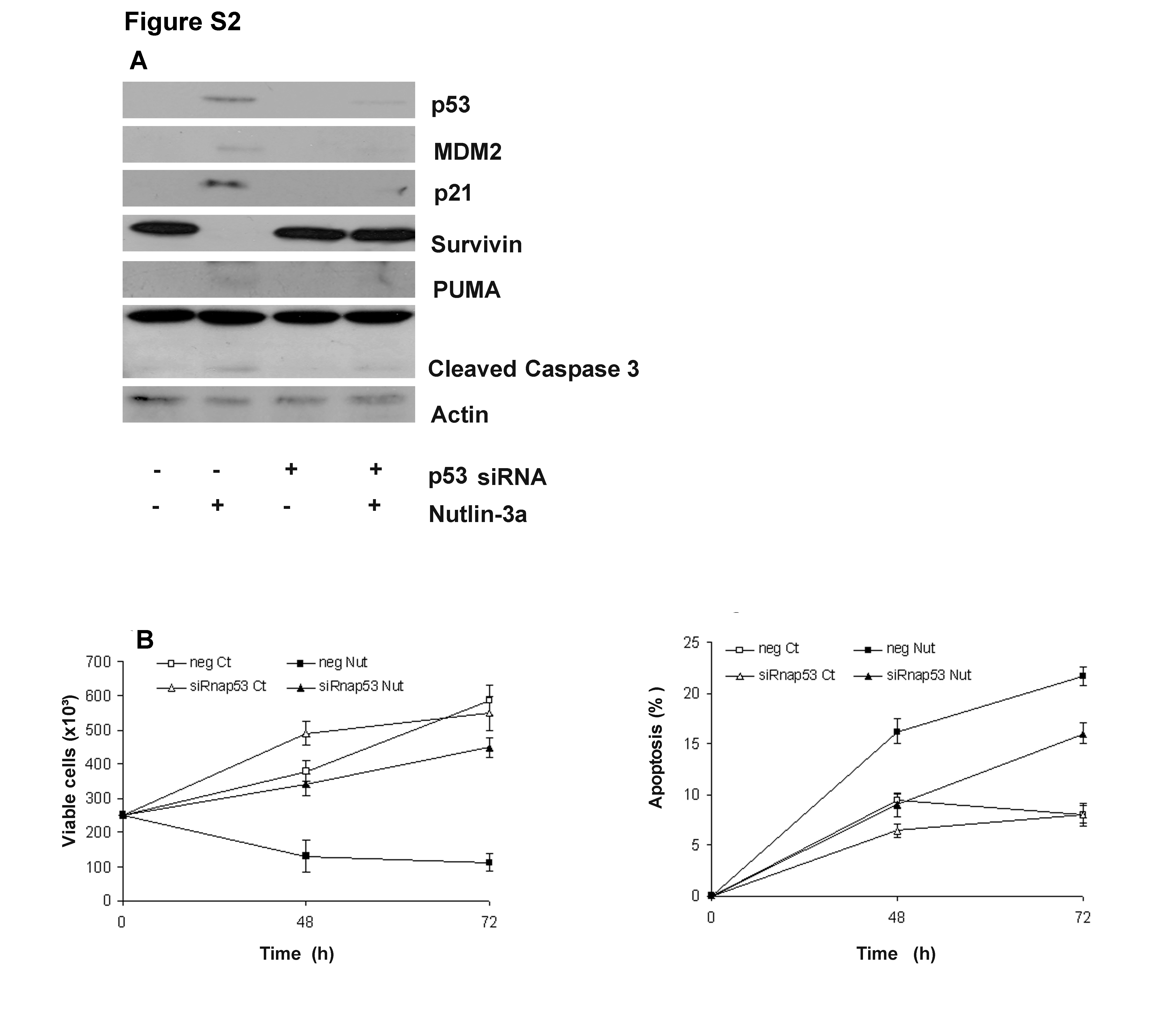

Supplement: Figure S2 — Silencing of p53 suppresses nutlin-3a cytotoxicity in wild-type p53 U87 cells. A, immunoblots demonstrating silencing of p53 protein in U87MG cells 48 hours after transfection and 72 hours after nutlin-3a treatment. Knockdown of p53 prevent U87MG cells to p53, p21, MDM2, Puma and cleaved caspase 3 proteins induction as well as Survivin down-regulation. Immunoblots are representative of at least three independent experiments. B, U87MG cells transfected either with p53 specific siRNA or negative-control siRNA and 6 hours later treated with nutlin-3a (10 µM) or DMSO (vehicle control; ct). The number of viable cells was counted with trypan blue exclusion assay at 48 and 72 hours. Points, average of three independent assays expressed as the mean ± sd. C, time course of nutlin-3a induced apoptosis in U87MG cells 6 hours after transfection and 48 and 72 h after treatment. Apoptosis was measured by surface Annexin V staining and flow cytometry as described in “Patients, materials and methods”. Average of a total of three independent assays ± sd. (TIF) [file pone.0018588.s002.tif]

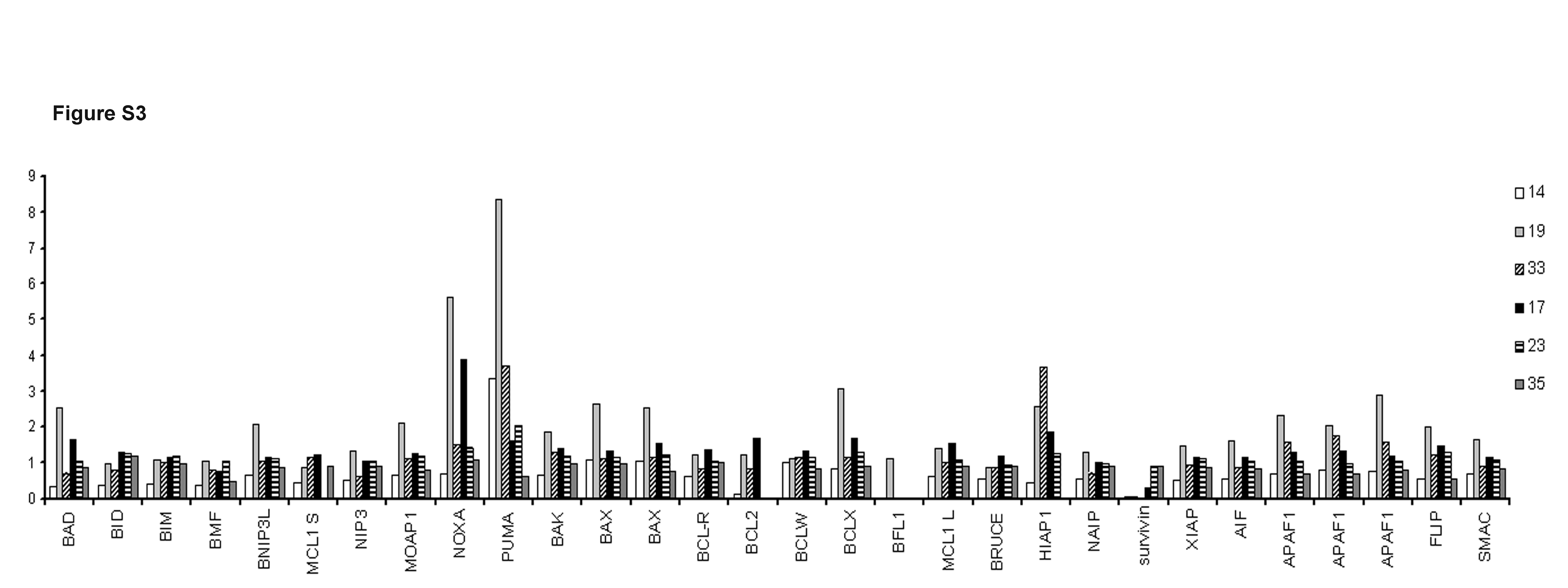

Supplement: Figure S3 — Primary cultured glioblastoma cells were treated with 10 µM nutlin-3a for 96 hours. Expression of apoptosis-related genes was analyzed by RT-MLPA as described in “Patients, materials and methods”. The results are shown as fold induction relative to untreated cells. (TIF) [file pone.0018588.s003.tif]
